# Genetic diversity of *Leptospira* isolates in Lao PDR and genome analysis of an outbreak strain

**DOI:** 10.1371/journal.pntd.0010076

**Published:** 2021-12-28

**Authors:** Linda Grillová, Matthew T. Robinson, Anisone Chanthongthip, Antony T. Vincent, Cecilia Nieves, Jan Oppelt, Jean-François Mariet, Céline Lorioux, Manivanh Vongsouvath, Mayfong Mayxay, Ooyanong Phonemeexay, Sayaphet Rattanavong, Koukeo Phommasone, Anousone Douangnouvong, David Šmajs, Frédéric J. Veyrier, Paul N. Newton, Mathieu Picardeau

**Affiliations:** 1 Biology of Spirochetes Unit, Institut Pasteur, Paris, France; 2 Department of Biology, Faculty of Medicine, Masaryk University, Brno, Czech Republic; 3 Lao-Oxford-Mahosot Hospital-Wellcome Trust-Research Unit (LOMWRU), Microbiology Laboratory, Mahosot Hospital, Vientiane, Lao People’s Democratic Republic; 4 Centre for Tropical Medicine & Global Health, Nuffield Department of Medicine, University of Oxford, Oxford, United Kingdom; 5 INRS-Centre Armand-Frappier Santé-Biotechnologie, Bacterial Symbionts Evolution, Laval, Canada; 6 Department of Pathology and Laboratory Medicine, Perelman School of Medicine, University of Pennsylvania, Philadelphia, United States of America; 7 Institute of Research and Education Development (IRED), University of Health Sciences, Ministry of Health, Vientiane, Lao People’s Democratic Republic; University of Minnesota, UNITED STATES

## Abstract

**Background:**

Although Southeast Asia is one of the most leptospirosis afflicted regions, little is known about the diversity and molecular epidemiology of the causative agents of this widespread and emerging zoonotic disease.

**Methodology/Principal findings:**

We used whole genome sequencing to examine genetic variation in 75 *Leptospira* strains isolated from patients in the Lao PDR (Laos) between 2006 and 2017.

Eleven serogroups from 4 *Leptospira* species and 43 cgMLST-defined clonal groups (CGs) were identified. The most prevalent CG was CG272 (n = 18, 26.8%), composed of *L*. *interrogans* serogroup Autumnalis isolates. This genotype was recovered throughout the 12-year period and was associated with deaths, and with a large outbreak in neighbouring Thailand. Genome analysis reveals that the CG272 strains form a highly clonal group of strains that have, for yet unknown reasons, recently spread in Laos and Thailand. Additionally, accessory genes clearly discriminate CG272 strains from the other *Leptospira* strains.

**Conclusions/Significance:**

The present study reveals a high diversity of *Leptospira* genotypes in Laos, thus extending our current knowledge of the pan- and core-genomes of these life-threatening pathogens. Our results demonstrate that the CG272 strains belong to a unique clonal group, which probably evolved through clonal expansion following niche adaptation. Additional epidemiological studies are required to better evaluate the spread of this genotype in Southeast Asia. To further investigate the key factors driving the virulence and spread of these pathogens, more intense genomic surveillance is needed, combining detailed clinical and epidemiological data.

## Author summary

Pathogenic *Leptospira* are the causative agents for leptospirosis, a neglected and emerging zoonosis occurring worldwide. In this study, we investigated the genetic diversity of *Leptospira* strains isolated from patients over a 12-year period in Lao PDR. Our genome analysis revealed a high diversity of *Leptospira* genotypes in this endemic country for leptospirosis, including a group of clonal strains which was responsible for a large outbreak in neighboring Thailand. Further progress in our understanding of the epidemiology of *Leptospira* circulating genotypes should contribute to the implementation of prevention and intervention measures to reduce the risk of leptospirosis transmission.

## Introduction

It is estimated that one million patients suffer severe leptospirosis each year with nearly 60,000 deaths, mostly in developing tropical countries [1]. The global burden of leptospirosis in terms of disability-adjusted life years (DALYs) is in the same range or even higher than for dengue, rabies, schistosomiasis, leishmaniasis and lymphatic filariasis [2]. It is very likely underestimated because of misdiagnosis and inadequate surveillance systems in place in most countries, particularly where other diseases with similar non-specific presentations, such as dengue and malaria, are prevalent. Leptospirosis is likely to become even more prevalent due to (i) global climate changes resulting in more frequent and severe flooding [3], and (ii) the growing population residing in urban slums [4].

Pathogenic *Leptospira* colonize the proximal renal tubules of reservoir hosts and are excreted through urine into the environment. Infections usually occur through contact with water or soil contaminated with the urine of infected animals. Leptospires are highly motile spirochetes that penetrate abraded skin and mucous membranes to rapidly disseminate hematogenously, causing fever, Weil’s disease or pulmonary hemorrhage syndrome [5].

*Leptospira* is a highly heterogeneous bacterial genus, divided into 64 species [6], 17 of which are potentially infectious to both humans and animals, and subdivided in nearly 300 serovars. However, for yet unknown reasons, a limited number of *Leptospira* serovars are much more likely to cause severe disease than others [7–9]. Recently, we provided insights into the virulence evolution of *Leptospira* spp. [6,10, 11], identifying a group of species among the pathogens/subclade P1 most often associated with severe infections [6]. Better understanding of the diversity of *Leptospira* strains is important to (i) identify strains or genotypes responsible for severe infections, (ii) evaluate the accuracy of current diagnostic tools and whole *Leptospira*-vaccines and (iii) develop control and prevention strategies for *Leptospira* serovars associated with particular animal reservoirs. For this purpose, many different molecular typing schemes have been developed, including Pulse-Field Gel Electrophoresis (PFGE) [12], Multi-Locus Variable Number Tandem Repeat (VNTR) analysis [13], and several Multi-Locus Sequence Typing (MLST) methods [14,15]. This diversity of typing methods applied to different sample sets has resulted in fragmentation of our epidemiological knowledge of leptospirosis. Recently, we proposed a universal core genome MLST (cgMLST) scheme that allows high-resolution genotyping of isolates across the entire *Leptospira* genus [16].

Leptospirosis is endemic in most countries of South and Southeast Asia [1,17–22] and these regions appear to have the highest global burden estimates of leptospirosis with an estimated 266,000 cases and 14,200 deaths annually [1]. Laos is a land-linked country of seven million people, bordering China, Vietnam, Cambodia, Thailand, and Myanmar, with limited information on the circulating *Leptospira* strains. Serological surveys in Laos showed evidence of past leptospiral infections in 19% to 45% of the population [23,24]. Pathogenic *Leptospira* spp. are one of the leading bacterial pathogens causing fever [24–26], central nervous system infections [27] and acute jaundice [28,29] in Laos. We isolated strains from patients in Laos over 12 years and conducted whole-genome sequencing of 68 *Leptospira* isolates along with 7 from Laos already described [30] to yield a better understanding of the evolutionary dynamics of *Leptospira*.

## Methods

### Ethics statement

The study protocols were approved by the National Ethics Committee for Health Research, Government of the Lao PDR (134/2007), the Ethical Review Committee of Research MoH (2000) and the Oxford Tropical Research Ethics Committee, UK (006–07 and 015–10) and were conducted in compliance with the Declaration of Helsinki. All participants provided written informed consent. Written consent was obtained from the parent/guardian of each participant under 16 years of age. In Lao heath care, people aged 16 years and above are considered adults for health care decisions. The approved Lao national ethics clearance for this study included that patients aged 16 years and above are considered adults and therefore parent/guardian consent was not required.

### Study design and study population

The isolates used here were from cultured blood clots left over after centrifugation to collect sera for aetiological investigations of the causes of fever. Blood clots from patients of any age and either sex admitted to Mahosot Hospital and Friendship Hospital, Vientiane City [31] with suspected community-acquired bacteremia were included provided they gave informed written consent (2006–2017). In addition, blood clots from inpatients and outpatients at Luang Namtha Provincial Hospital and Salavan Provincial Hospital (2010–2015) were included if they were aged 5–49 years, gave written informed consent, were eligible for malaria rapid diagnostic testing or microscopy by Lao national guidelines, had no obvious causes of fever (abscess or severe diarrhoea) with fever <8 days, and an admission tympanic temperature of >38 C [25]. History and clinical examination were recorded on standardised forms. Over the 12 years 36,495 patient blood samples were cultured for *Leptospira*, and a total of 158 isolations were made, with 75 successfully subcultured for this analysis. Epidemiological, clinical, and outcome features of the patients are shown in S1 Table.

### Culture and *Leptospira* strains

Whole non-anticoagulated blood samples were collected from inpatients with suspected leptospirosis during 2006–2017 at the four hospitals (Mahosot Hospital and Friendship Hospital in Vientiane, Luang Namtha Provincial Hospital, and Salavan Provincial Hospital) [25,31]. Whole blood was centrifuged, serum removed and clots were incubated in Ellinghausen, McCullough, Johnson and Harris (EMJH) medium [32,33] overnight. The EMJH medium was then separated from the blood clot and incubated at 30°C for up to 12 weeks with checks for growth by dark-field microscopy every 2 weeks at the Microbiology Laboratory, Mahosot Hospital. Serotyping was performed at the National Reference Centre for Leptospirosis (Institut Pasteur, Paris, France) as previously described with a panel of rabbit antisera representing 24 serogroups [34,35].

### Whole-genome sequencing

Illumina sequencing was performed from extracted genomic DNAs of 68 exponential-phase cultures using a MagNA Pure 96 Instrument (Roche, Meylan, France). Next-generation sequencing (NGS) was performed using Nextera XT DNA Library Preparation kit and the NextSeq 500 sequencing systems (Illumina, San Diego, CA, USA) at the Mutualized Platform for Microbiology (P2M) at Institut Pasteur. CLC Genomics Workbench 9 software (Qiagen, Hilden, Germany) was used for analyses. The generated contig sequences together with the sample metadata are available in BIGSdb hosted at the Institut Pasteur (https://bigsdb.pasteur.fr/leptospira/). We also downloaded 7 additional genome sequences of *Leptospira* isolates from Laos from the NCBI database (S1 Table). These isolates originated from the same sources as detailed above [30]. Genome features of the 75 isolates are indicted in S1 Table.

PacBio SMRT (Pacific Biosciences) sequencing was also performed for *L*. *interrogans* serogroup Autumnalis strain id779, a representative Laos strain belonging to the clonal group 272. Genomic DNA was extracted from a 35 ml culture with the Genomic tip 100 g kit (Qiagen, Hilden, Germany) according to manufacturer protocols. PacBio sequencing was performed at the Génome Québec Innovation Centre (McGill University, Montreal, Canada) using a Pacific BioScience RS II system. The sequencing reads were *de novo* assembled using Unicycler [36]. The complete genome of *L*. *interrogans* serogroup Autumnalis strain id779 can be visualized in the MicroScope Platform (https://mage.genoscope.cns.fr/microscope/home/index.php). Sequences were deposited in GenBank under accession number SAMN18642399.

### Reconstruction of complete reference genomes of *L*. *interrogans*

Quality of the sequencing reads was checked by FastQC (v0.11.5) (https://www.bioinformatics.babraham.ac.uk/projects/fastqc/), and the potential contaminants were scanned by Kraken2 (v2.0.7-beta) [37]. Illumina reads were digitally normalized to an average depth of 100x using bbnorm.sh (v38.67) (https://sourceforge.net/projects/bbmap/) before the assembly to avoid assembling errors. Reads with an apparent depth of 5x were discarded. The normalized Illumina reads were corrected by SPAdes (v3.13.1) [38].

PacBio reads were filtered by Filtlong (v0.2.0) (https://github.com/rrwick/Filtlong) and the genome assembly was performed using Raven (commit 8632651) [39] with the default settings. Raven performs two rounds of Racon [40] polishing by default. The resulting assembly was further polished with two additional rounds of Pilon (v1.23) [41] using the Illumina reads. The PacBio reads correction by Illumina reads was performed by FMLRC (v1.0.0) [42]. Potential missassemblies and the overall assembly quality was continuously evaluated by ideel (commit 78bb26c) (https://github.com/phiweger/ideel), *FRCbam* (v1.3.0) [43], Samtools stats (v1.9) [44], and MUMmer dotplot (v4.0.0beta2) [45]. Correct assembly scaffolds orientation and circular chromosomes starts were determined by Circulator (v1.5.6) [46] and BLAST (v2.9.0+) [47]. Illumina-only assembly (SPAdes (v3.13.1) [38], k-mer settings 27,47,63,77,87,97,105,113,119,125; minimal coverage 5x) was used to check for missing short scaffolds in the PacBio assembly. For the final assembly correction step, Illumina reads were mapped by bwa-mem (v0.7.17-r1188) [bwa] and variants with 100% occurrence as called by FreeBayes (v1.3.1) (https://arxiv.org/abs/1207.3907v2) were manually inserted into the assembly.

The reference-guided assembly of other samples was performed as described previously [48]. The *de novo* assembled genome was used as a reference in this step.

### Genome analysis

The Percentage Of Conserved Proteins (POCP) values were determined using GET_HOMOLOGUES version 08042020 [49] and the OMCL algorithm [50]. Functional annotation was carried out using eggNOG-mapper v2 (http://eggnog-mapper.embl.de) [51]. Representativeness of each functional category found among different clonal groups was expressed as a proportion of the total number of predicted proteins in each genome. An unpaired parametric t-test was used to compare the two independent groups. The core and pan genome of *Leptospira* was also evaluated using the same tool. Graphical representation of the results as well as statistical analysis were performed with GraphPad Prism 6. Circular maps were generated using CGview [52].

Core genome Multi-Locus Sequence (cgMLST) profiles were determined using BIGSdb as described previously [16]. Briefly, 545 core genomes were extracted, concatenated and analyzed in order to determine the cgST (core genome Sequencing Type) and cgMLST CGs (cgMLST Clonal Groups). A CG is defined as a group of cgMLST allelic profiles differing by no more than 40 allelic mismatches out of 545 gene loci. Phylogenetic trees were generated with MEGA 6 [53] using the Tamura Nei model and 100 pseudorandom bootstrap replicates. To compare sequencing data obtained from Laos with all available sequencing data obtained from the neighboring countries of Southeast Asia, we extracted the six genes (*glmU*, *pntA*, *sucA*, *tpiA*, *pfkB*, *mreA*, *caiB*) that are part of the MLST 1 scheme [30] from our raw data and enhanced our sample set by additional sequences stored at pubMLST (https://pubmlst.org/) database (S2 Table). The open-access and curated MLST (https://pubmlst.org/) and cgMLST (https://bigsdb.pasteur.fr/) databases for *Leptospira* were used as the source of strain metadata (reservoir, geographic location, etc).

The population dynamic of identified genotypes was shown by Muller diagram generated in R (version 3.6.1.) using the MullerPlot package [54]. The statistical interference was calculated in R (version 3.6.1).

### PCR and molecular typing

PCR was performed using the published primers for amplifying *lfb*1[55], *lipL*32 [56] and *sec*Y [57,58] (S3 Table) under the following conditions: 95°C (1 min); 94°C (30 s), 55°C (30 s), and 72°C (1 min) for 35 cycles; with the final extension at 72°C for 10 min. Each PCR mixture contained 1U of GE Healthcare Recombinant Taq DNA Polymerase (Scientific Laboratory Supplies, Nottingham, UK), 5μM of dNTP, 25mM of MgCl2, 5 μl of 10x buffer, 10μM of each primer, and 5 μl of DNA extract from culture isolates. Sanger sequencing of PCR products was performed by Eurofins Genomics Germany GmbH (Ebersberg, Germany) and the sequence analyses were performed with Lasergene software (DNASTAR v. 7.1.0.; DNASTAR, Madison, WI).

## Results

### *Leptospira* strains isolated from patients

We used 75 genomes from *Leptospira* clinical isolates recovered from patients between 2006 and 2017 to assess the genetic diversity of circulating strains in Lao PDR. Over 12 years (2006–2017), 36,495 patient blood samples were cultured for *Leptospira* during investigation of the aetiology of fever in Laos. A total of 158 isolations were obtained, and 75 were analysed in this study. The majority of infections among the 75 patients occurred during the warm wet season, i.e. June to October (68% of patients). *Leptospira* isolates were collected from patients living in the capital (56%) and in six provinces: Vientiane (10, 7%), Luangprabang (11.3%), Saravan (6.5%), Luangnamtha (3%), Bolikhamsai (1.7%) and Bokeo (1.7%); with information unavailable for 10 isolates. Genome analysis of the 75 *Leptospira* isolates indicated that all belonged to the pathogenic species *L*. *interrogans* (n = 61), *L*. *weilii* (n = 9), *L*. *borgpetersenii* (n = 3) and *L*. *kirschneri* (n = 2). Serogroup Autumnalis was the main serogroup identified (n = 22, 29.3%). Other *Leptospira* serogroups identified were Bataviae (n = 9, 12%), Canicola (n = 9, 12%), Grippotyphosa (n = 9, 12%), Celledoni (n = 4, 5.3%), Pomona (n = 4, 5.3%) with serogroups Australis, Hebdomadis, Icterohaemorrhagiae, Javanica, Mini and Pyrogenes, represented by three strains or less (S1 Table).

Among the 75 patients, there was a higher proportion of males (71.2%) and the median (range) age was 30.0 (4–73) years. The Lao Loum, who represents 75% of the population of Laos, was the main ethnic group represented (88.4%), followed by Lao Thung (5.8%) and Lao Sung (5.8%) (S1 Table). Demographic, clinical and occupational characteristics of the study population are shown in Tables 1 and S1. Patients presented with median (range) of 3.9 (1–7) days of acute illness and most exhibited fever, headache and myalgia. Five patients died. Females had a higher frequency of deaths (33.3% of females and 7.1% of males, p = 0.0228). However, women affected by leptospirosis were older (median (range) age 34 (10–65) years) than males (mean (range) age 21.5 (4–73) years) (p = 0.0271). The vast majority of patients (95.5%) reported that they had seen rodents in the last 2 weeks before attending hospital (S1 Table).

**Table 1:** Admission clinical characteristics of *Leptospira* spp. culture positive patients.

Variable (%)	Data available (n = 75)	Patients (%)
Fever	72	72 (100)
Rigors	70	40 (57.1)
Headache	71	63 (88.7)
Arthralgia	70	42 (60.0)
Myalgia	73	55 (75.3)
Nausea	70	20 (28.6)
Vomiting	71	21 (29.6)
Diarrhea	71	15 (21.1)
Abdominal pain	70	13 (18.8)
No. of deaths	37	5 (13.5)

### Clonal groups and clusters of Lao *Leptospira* isolates

**Fig 1:**
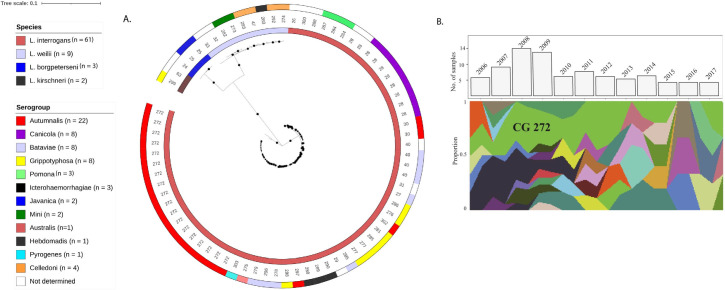
The majority of Laos strains belong to the clonal group 272 of *L*. *interrogans* sg Autumnalis. A. Maximum-likelihood phylogeny based on the variable sites of the cgMLST scheme consisting of 545 core genes showing the distribution of species, cgMLST clonal groups, and serogroups circulating in Laos during 2006–2017 (n = 75, S1 Table). The samples were divided into 43 cgMLST clonal groups belonging to 12 different serogroups; white color indicates that the serogroup is unknown or undetermined. Bootstrap values are shown with the size of circles in the middle of branches, bootstraps lower than 80 are not shown. Species and serogroups are given by the color codes (from inner to outer circles) and cgMLST clonal groups as numbers. B: Muller diagram showing distribution of clonal groups in Laos during 2006–2017. The most prevalent clonal group, CG272, is indicated. The bar chart is showing the number of samples sequenced in each year. The population dynamic of genotypes was produced in R using the MullerPlot package [54].

To investigate the genetic diversity of the 75 clinical isolates, we used a core genome MLST (cgMLST) scheme based on 545 genes [16]. cgMLST divided our sample set into four *Leptospira* species (above) and 43 clonal groups (CGs) (Fig 1A). The most prevalent CG was CG272 (n = 18, 26.9%), a novel CG composed of isolates belonging to *L*. *interrogans* serogroup Autumnalis. CG28 (n = 10, 13.3%), corresponding to *L*. *interrogans* serogroup Canicola, has been isolated from patients, rats, dogs, pigs and calves suggesting that this genotype is not restricted to a specific reservoir but is a widespread generalist genotype. CG40 (n = 4, 5.3%), composed of strains of *L*. *interrogans* serogroup Bataviae, has been described from a patient in Thailand. The remaining CGs are represented by less than three strains and they have not been found in other regions of the world, with the exception of CG25 (n = 2) which has been already isolated in Malaysia and Thailand.

Among the Lao clinical isolates, most (37/43, 86%) CGs were detected only in a particular year, while other CGs declined and disappeared over a period of years. The only CG detected in almost every year (10/12 years) was CG272, associated with *L*. *interrogans* serogroup Autumnalis (Fig 1B).

Patient death occurred with CG272 (*L*. *interrogans* serogroup Autumnalis, n = 2), CG25 (*L*. *borgpetersenii* serogroup Javanica, n = 1), CG290 (*L*. *interrogans* serogroup Icterohaemorrhagiae, n = 1), and CG300 (*L*. *interrogans*, unknown serogroup, n = 1).

**Fig 2:**
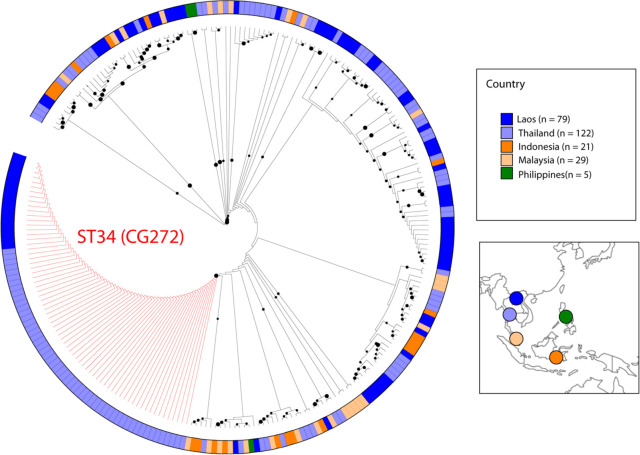
Prevalence of the ST34-like strains in Laos and neighbouring countries. Phylogenetic relatedness of pathogenic *Leptospira* strains (n = 258) circulating in Southeast Asia. Maximum-likelihood phylogeny based on 994 SNVs found in 3,113 bp-long concatenated sequences of *glm*U, *pnt*A, *suc*A, *tpi*A, *pfk*B, *mre*A *and cai*B loci characterized by MLST1 [30] together with the samples analyzed in this study. Isolates belonging to the ST34 (CG272) are highlighted in red. Metadata associated with the isolates not listed in S1 Table are provided in S2 Table. The base layer of the map is from outline-world-map.com.

Since there are few genomes of *Leptospira* strains isolated in Southeast Asia, we translated the whole genome data of these Lao clinical isolates into the widely used MLST 1 scheme [30]. We then compared the genetic data obtained in Laos with available Sequence Types (ST) in neighboring countries by MLST (Fig 2 and S2 Table). Strains belonging to CG272 were clustered by MLST with ST34 isolates forming the largest cluster (71 out of 258; 27.5%). ST34 strains were described as responsible for an outbreak of leptospirosis in northeast Thailand between 1999 and 2003 [59], and also isolated from bandicoot rats (*Bandicota indica* and *Bandicota savilei*) in Thailand [59].

### Genomic features of the outbreak strain CG272 / ST34 and comparative genomics with other *L*. *interrogans* strains

**Fig 3:**
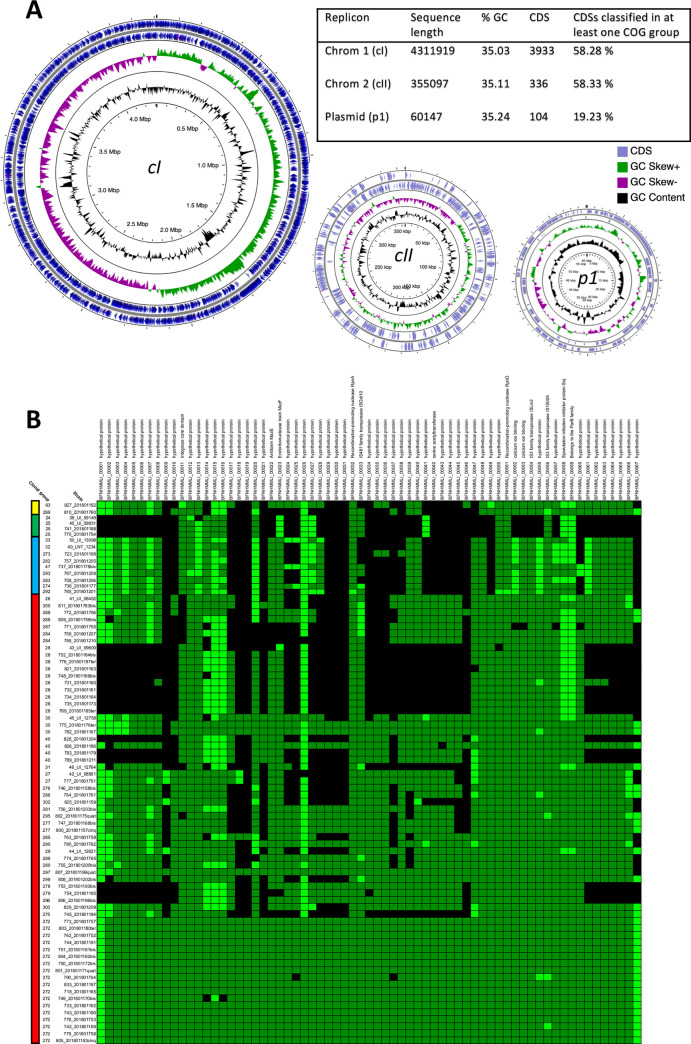
Genome features of *L*. *interrogans* sg Autumnalis strain id779. A. Circular maps and genome features of the three replicons of *L*. *interrogans* sg Autumnalis strain id779. B. Heatmap showing absence/presence of plasmid genes of *L*. *interrogans* sg Autumnalis strain id779 in other Laos strains. The strains were ordered according to the phylogenetic tree shown in Fig 1A. The heatmap was obtained by doing a TBLASTN analysis of the sequences of the plasmid against the genomic sequences of the strains. A gene was considered to be present if the hit had an e-value greater than 1e-10 and 50% similarity. The light green represents sequences with a similarity percentage between 50% and 79%, while the dark green represents sequences with a similarity percentage of 80% to 100%. Color bar on the left corresponds to: red, *L*. *interrogans*; blue, *L*. *weilii*, green, *L*. *borgpetersenii*; yellow: *L*. *kirschneri*.

As there was no whole genome sequence of the ST34 outbreak strain available in public databases we obtained the complete genome of one representative ST34/CG272 Laos strain using the PacBio single-molecule real-time (SMRT) sequencing method. The genome of *L*. *interrogans* serogroup Autumnalis strain id779 from a student who died of leptospirosis (S1 Table) was composed of two chromosomes and one plasmid with the total length of 4,727,163 bp and a total number of 4,373 CDSs (Fig 3A). This isolate possesses an array of known virulence genes [60] including genes encoding for more than 100 membrane-associated lipoproteins such as LipL32 and LigA/B, 17 Leucine-Rich proteins, 5 hemolysins, 3 sphingomyelinases, etc. Moreover, this strain shares more than 85% (minLrap ≥ 0.8; maxLrap ≥ 0; Identity ≥ 35%) of the CDSs found in the highly virulent strains *L*. *interrogans* serovar Copenhageni strain Fiocruz L1-130 and *L*. *interrogans* serovar Manilae strain UP-MMC-NIID LP, as determined using the MaGe Web interface (http://www.genoscope.cns.fr/agc/mage). Interestingly, strain id779 contains a 60-kb circular plasmid with most of the genes (>80%) encoding hypothetical proteins of unknown function (Fig 3A). Analysis of the presence of these plasmid genes in other Laos strains revealed that this plasmid is highly conserved within the CG272 strains and also present in other Autumnalis (CG297 and CG30) and Icterohaemorrhagiae (CG290, CG280, CG289) isolates (Fig 3B).

In order to investigate the genes that may be advantageous for environmental adaptation, host transmission, persistence or virulence of these ST34/CG272 strains compared with other strains, we analyzed and compared the deducted proteome of the two groups of *L*. *interrogans* strains (CG272 vs non-CG272 strains). To examine this, we first identified the percentage of conserved proteins (POCP) [61] of *L*. *interrogans* strains. The complete set of POCP values for all *L*. *interrogans* isolates are given in S4 Table. The POCP values for pairwise comparisons of each of the CG272 strains are ≥92%, confirming that they form a homogenous group of closely related strains. Other *L*. *interrogans* strains exhibited POCP ranging from 53–90%, when compared with *L*. *interrogans* serogroup Autumnalis strain id779. Interestingly, higher genetic relatedness (POCP values ranging from 88–90%) was found between *L*. *interrogans* serogroup Autumnalis strain id779 and *L*. *interrogans* strains belonging to serogroups Grippotyphosa, Canicola, Autumnalis and Australis (S4 Table).

Among the *Leptospira* strains from Laos, we identified a pangenome of 11,748 unique protein-coding sequences. A large majority of genes (81%) in the pangenome are part of the accessory genome, which comprised the shell and cloud genes. Similarly, the pan-genome analysis of *L*. *interrogans* non-CG272 strains shows a strong enrichment (≈5X) of gene clusters that are unique to one species (5681) as compared to gene clusters in the core genome (1211). In contrast, the core genome of *L*. *interrogans* CG272 strains accounted for 72% of the genes present in the pangenome (S1 Fig).

**Fig 4:**
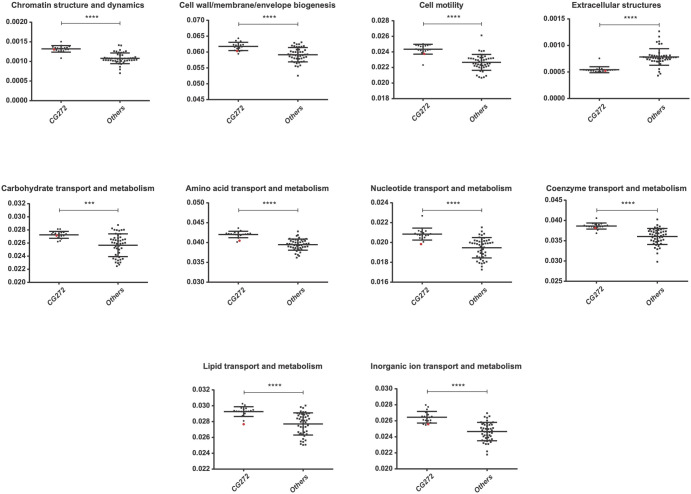
Distribution in functional categories of the predicted CDSs (%). Each dot represents the value for a specific genome after normalizing by its predicted total number of proteins. The genome-specific values of the *L*. *interrogans* CG272 strains and *L*. *interrogans* non- CG272 strains are presented. The genome of *L*. *interrogans* sg Autumnalis strain id779 is represented as a red dot. Only functional categories showing significant difference between the different groups are shown (*** P < 0.001, **** P < 0.0001).

As a second approach to explore signs of adaptation at the genomic level, orthology prediction was used to analyze the functional annotation of the genomes included in this study. Clusters of orthologous groups (COGs) of proteins were generated as a result (S5 Table). COG categories present in CG272 strains were contrasted against those of other clonal groups considered as a whole. In order to eliminate interspecies-related variability, only *L*. *interrogans* strains were included. Overall, those categories related to information storage and processing such as replication, transcription, or translation, among others, show little or no difference between groups. The only exception within this category is "Chromatin structure and dynamics", which shows a major difference with highest representation in CG272 strains (Fig 4). Interestingly the categories comprising transport and metabolisms of diverse molecules were over-represented in the CG272 strains suggesting an enrichment in metabolism processes.

**Fig 5:**
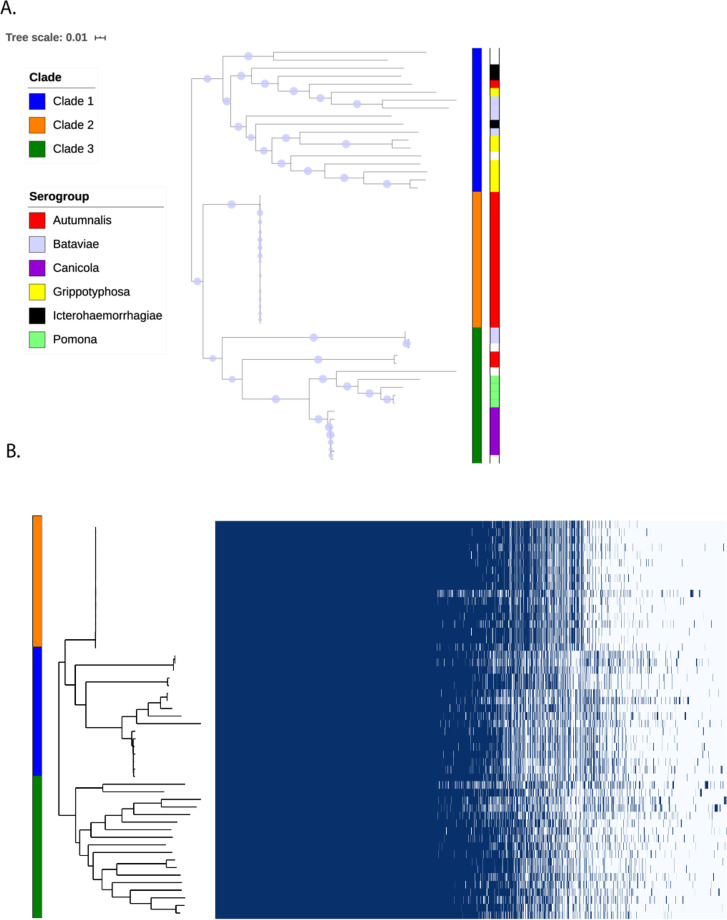
The *Leptospira* ST34-like strains have limited genetic variation. A: ML phylogeny based on whole genome sequences of *L*. *interrogans*. Bootstrap values are shown with the size of circles in the middle of the branches, bootstraps lower than 70 are not shown. Clade and serogroup names are given by the color codes (from inner to outer strips). White color indicates that the information was not available. B: Matrix showing absence or presence of 5,543 genes found in the pangenome of *L*. *interrogans* strains. Clade names are given by the color codes.

To further resolve population structure of pathogenic *Leptospira* strains in Laos, we have reconstructed nearly complete genomes of *L*. *interrogans* strains investigated in this study using the complete genome of *L*. *interrogans* serogroup Autumnalis strain id779 as a reference (S6 Table). While achieving higher resolution, the ML phylogeny based on whole genome sequences (Fig 5A) recapitulated the branching of ML phylogeny based on the 545 core genes used in cgMLST (Fig 1A). *L*. *interrogans* strains were clustered in three clades with CG272 strains (n = 17) representing a separate monophyletic group (Fig 5A). The average nucleotide distance of CG272 isolates forming clade 2 was surprisingly low (0%) in comparison to the average nucleotide distance within the isolates belonging to the clade 1 and clade 3 (0.4% and 0.3%, respectively). In fact, there were only 62 SNPs across the whole genome sequences found among the 17 strains belonging to CG272/clade 2 isolated over a period of nearly 12 years. As revealed by the minimum spanning tree (GrapeTree), the CG272 was a dominant central genotype forming a star-like topology from which the other genotypes radiated (S2 Fig and S7 Table).

In order to identify markers that could discriminate between ST34/CG272 isolates and other circulating isolates, we tested the most frequently used diagnostic and/or typing PCR assays (S3 Table). *In silico* PCR showed that all primers targeting *lfb*1, *lipL*32 and *sec*Y are able to specifically bind to all isolates in this study with at most 2 mismatches per primer sequence. To assess the resolution power of the assays, we compared the ML phylogeny build from the sequences of PCR products to the core genome phylogeny. Sequencing of the PCR products of *lfb1*, *lipL32* and two *secY* assays was able to distinguish 32%, 7%, 32.5% and 49% clonal complexes identified using cgMLST, respectively. Most importantly, sequencing the 549 bp long *secY* DNA fragment [57] with the highest resolution power (49%) was the only assay able to straightforwardly distinguish the CG272 strains from other strains.

## Discussion

We used genome sequences of 75 *Leptospira* isolates from patients in Laos and metadata associated with these isolates. This is, to our knowledge, the largest genomic study that investigates the pathogenic *Leptospira* isolates circulating in a single country. We also included MLST data of clinical strains from other SE Asian countries (Thailand, Indonesia, Philippines, Malaysia) to explore the underlying diversity of *Leptospira* present and to understand the dynamics of epidemic and endemic *Leptospira*.

Core genome Multi-locus Sequence Typing (cgMLST) of the 75 strains revealed 43 different core genome clonal groups (cgCGs), revealing a high diversity of *Leptospira* genotypes in Laos. As described [16], one serogroup can be sub-divided in distinct CG but strains that are part of the same CG belong to the same serogroup. Among the 43 cgCGs, only 3 (CG272, n-18; CG28 = 10; and CG40 = 4) are composed of more than 3 strains. Analyses to identify associations of *Leptospira* genotypes to particular epidemiological variables, and specifically to test whether some genotypes are predictors of disease outcomes, cannot be performed with such a small sample size. The analysis of a larger number of strains would have more power to identify associations with clinical characteristics. Multiple definitions are currently in use for severe infections [7,62–65] and a consensus definition would help in finding associations between clinical severity and genotypes. Additional information such as the epidemiology of reservoir hosts, modes of transmission and patient comorbidities and treatment would also facilitate more wide-ranging analysis.

We show that a single clone of *L*. *interrogans* serogroup Autumnalis, previously identified as ST34 in Thailand [59], was responsible for a significant proportion of infections in Laos between 2006 to 2017. This group of strains has been predominant in Thailand and a large outbreak of ST34 strains was reported between 1998 and 2003 but investigations did not find any specific epidemiological factor linked to this outbreak [59]. Whether ST34 was present in Laos before 2006 is unknown. An examination of the fitness of this outbreak strain in comparison with non-sympatric outbreak strains in different environmental conditions (pH levels, temperatures, water sources) did not find any significant difference [66]. Several genomic features suggest that ST34/CG272 strains are clinically important clones that require more attention: i.) CG272 was a dominant central genotype forming a star-like topology from which the other genotypes radiated suggestive of clonal expansion of these strains, ii.) CG272 was the only clonal group detected in all but 2 years, iii.) CG272 form a well-defined monophyletic clade and these strains are highly clonal (there was only 62 SNPs found across the 17 genomes over a period of nearly 12 years), and finally v.) the average nucleotide distances and POCP values within CG272 were low compared to other phylogenetic clades. The CG272 is therefore a clinically important and highly-clonal sublineage of *L*. *interrogans* in this region. The low genome diversity among CG272 strains may reflect adaptation of this clonal group to a specific niche such as the bandicoot rats which were identified as a likely maintenance host of ST34 [59]. As a note, the *secY* allele of ST34 had also been recovered from a wild mouse, *Mus cookie*, in northern Thailand [67]. On the contrary, strains occupying different niches such as the non-CG272 isolates should experience a larger diversity of selection pressures, which will drive selection for increased genome diversity [68].

Although the mechanisms of pathogenesis of pathogenic *Leptospira* spp. is not completely understood, virulence-associated factors have been identified, including motility, adhesion, stress response, and evasion of immune response [69]. To further investigate if ST34/CG272 strains possess genes that may confer a phenotypic advantage over other *L*. *interrogans*, we looked at genetic differences between Lao ST34/CG272 strains and non-ST34/CG272 strains. CG272 strains had an enrichment of different COGs, mostly associated with metabolism processes, which may play important roles in the adaptation of CG272 strains to specific ecological niches. The presence of a 60 kb plasmid encoding for a large proportion of hypothetical proteins was not specific to CG272 strains as it was also found across different CGs and serogroups. We failed to identify any other accessory genomic elements which might have influenced the spread of ST34/CG272 strains. We are thus far from understanding the precise nature of this clonal success.

Concerted control efforts targeting CG272/ST34 isolates specifically could reduce epidemic and endemic risks of leptospirosis in Southeast Asia. *Leptospira* are fastidious and slow-growing bacteria and culture isolation from biological samples is still challenging. Here, we show that *secY* can be used as a discriminative marker to identify CG272/ST34 isolates from other circulating strains. *Leptospira* genotyping directly on biological samples should allow the epidemiological follow-up of circulating strains as previously shown in leptospirosis patients in French Polynesia [70]. Large-scale epidemiological studies in Laos and neighboring countries should better identify the prevalence over time and the geographic spread of CG272/ST34 isolates in both patients and potential reservoir hosts. *Leptospira* genotyping should also contribute to identifying the extent and mode of transmission of epidemic clone(s) in case of outbreaks and the impact of control interventions on disease transmission.

In conclusion, genome analysis associated with detailed epidemiological and clinical data should lead to major insights into the evolution, biology and pathogenesis of this emerging pathogen. Whole-genome based typing should become available as a routine tool because of the continued decrease in costs, thus improving surveillance methodology and outbreak investigations as it has for the COVID-19 pandemic. Although the isolation of *Leptospira* strains from biological samples remains challenging, recent advances such as the use of a new cocktail of antibiotics [71] and development of a novel culture medium for fastidious *Leptospira* [72] should facilitate expanded culture isolation.

## Supporting information

S1 Table *Leptospira* strains isolated in Laos examined in this study. Genome features and the patients epidemiological clinical outcome data are indicated.
(XLSX)

S2 Table List of isolates from Southeast Asia used for MLST scheme 1 and corresponding metadata. (XLSX)

S3 Table The Percentage Of Conserved Proteins (POCP) values of *L*. *interrogans* strains. The values of POCP were calculated by GET_HOMOLOGUES version 3.3.4 using the OMCL algorithm.
(XLSX)

S4 Table COG categories of genes in the *L*. *interrogans* genomes. From the files generated by eggNOG-mapper v2, the columns corresponding to "gene name" and "COG categories" for each *L*. *interrogans* genome were copied into a new Excel file named "All_genomes_COG_analysis.xlsx". The information for each genome is contained in separate sheets, named after the corresponding genome. The comparative analysis was focused on finding differences in categories related to metabolic pathways, since most of them showed highly significant differences among the different groups (CG272 vs Others): [G] Carbohydrate transport and metabolism, [E] Amino acid transport and metabolism, [F] Nucleotide transport and metabolism, [H] Coenzyme transport and metabolism, [I] Lipid transport and metabolism, [P] Inorganic ion transport and metabolism. Therefore, genes related to these pathways were filtered, and then the list of genes for each genome was compared using the online tool https://www.molbiotools.com/listcompare.html.
(XLSX)

S5 Table *L*. *interrogans* isolates with resolved near complete genomes/. Statistics from reference-guided Illumina reads mapping.
(XLSX)

S6 Table *L*. *interrogans* strains isolated from human patients in Southeast Asia (n = 81) during 1905–2017 (See S2 Fig). (XLSX)

S7 Table The most frequently used diagnostic and/or typing PCR assays. (DOCX)

S1 Fig Pan-genome distribution in four categories (cloud, shell, soft core and core) for *Leptospira* strains from Laos. Gene frequency plot showing the frequency of genes within the *Leptospira* strains. Analyses done with GET_HOMOLOGUES showing the U-shaped distribution of pan-genome from the three groups of strains: *L*. *interrogans* CG272 strains (CG272), *L*. *interrogans* non-CG272 strains (Others) and all the *Leptospira* strains (complete).
(DOCX)

S2 Fig Core genes of all available *L*. *interrogans* strains isolated from human patients in Southeast Asia (n = 81) during 1905–2017. Minimum spanning tree was created using GrapeTree for visualization of core genomic relationships [1]. Every tree node represents a core genome of a single sample, the cgMLST clonal groups are indicated by the numbers inside the tree nodes and the geographic origin is determined by colors. Strains are listed in S6 Table. The base layer of the map is from outline-world-map.com.
(DOCX)

## References

[1] Costa F, Hagan JE, Calcagno J, Kane M, Torgerson P, Martinez-Silveira MS, et al. Global Morbidity and Mortality of Leptospirosis: A Systematic Review. PLoS Negl Trop Dis 2015;9:e0003898. doi: 10.1371/journal.pntd.0003898 26379143 PMC4574773

[2] Torgerson PR, Hagan JE, Costa F, Calcagno J, Kane M, Martinez-Silveira MS, et al. Global Burden of Leptospirosis: Estimated in Terms of Disability Adjusted Life Years. PLoS Negl Trop Dis 2015;9:e0004122. doi: 10.1371/journal.pntd.0004122 26431366 PMC4591975

[3] Munoz-Zanzi C, Groene E, Morawski BM, Bonner K, Costa F, Bertherat E, et al. A systematic literature review of leptospirosis outbreaks worldwide, 1970–2012. Rev Panam Salud Publica. 2020;44:e78. doi: 10.26633/RPSP.2020.78 32684917 PMC7363284

[4] Costa F, Carvalho-Pereira T, Begon M, Riley L, Childs J. Zoonotic and Vector-Borne Diseases in Urban Slums: Opportunities for Intervention. Trends Parasitol. 2017;33:660–2. doi: 10.1016/j.pt.2017.05.010 28625886

[5] Ko AI, Goarant C, Picardeau M. *Leptospira*: the dawn of the molecular genetics era for an emerging zoonotic pathogen. Nat Rev Microbiol 2009;7:736–47. doi: 10.1038/nrmicro2208 19756012 PMC3384523

[6] Vincent AT, Schiettekatte S, Goarant C, Neela VK, Bernet E, Thibeaux R, et al. Revisiting the taxonomy and evolution of pathogenicity of the genus *Leptospira* through the prism of genomics. PLoS Negl Trop Dis. 2019;13:e0007270. doi: 10.1371/journal.pntd.0007270 31120895 PMC6532842

[7] Tubiana S, Mikulski M, Becam J, Lacassin F, Lefèvre P, Gourinat AC, et al. Risk factors and predictors of severe leptospirosis in New Caledonia. PLoS Negl Trop Dis 2013;7:e1991. doi: 10.1371/journal.pntd.0001991 23326614 PMC3542117

[8] Taylor AJ, Paris DH, Newton PN. A Systematic Review of the Mortality from Untreated Leptospirosis. PLoS Negl Trop Dis 2015;9:e0003866. doi: 10.1371/journal.pntd.0003866 26110270 PMC4482028

[9] Hochedez P, Theodose R, Olive C, Bourhy P, Hurtrel G, Vignier N, et al. Factors Associated with Severe Leptospirosis, Martinique, 2010–2013. Emerg Infect Dis 2015;21:2221–4. doi: 10.3201/eid2112.141099 26583702 PMC4672444

[10] Fouts DE, Matthias MA, Adhikarla H, Adler B, Berg DE, Bulach D, et al. What Makes a Bacterial Species Pathogenic?: Comparative Genomic Analysis of the Genus *Leptospira*. PLoS Negl Trop Dis. 2016;10:e0004403. doi: 10.1371/journal.pntd.0004403 26890609 PMC4758666

[11] Thibeaux R, Iraola G, Ferrés I, Bierque E, Girault D, Soupé-Gilbert ME, et al. Deciphering the unexplored *Leptospira* diversity from soils uncovers genomic evolution to virulence. Microb Genom 2018;4(e000144). doi: 10.1099/mgen.0.000144 29310748 PMC5857368

[12] Herrmann JL, Bellenger E, Perolat P, Baranton G, Saint Girons I. Pulsed-field gel electrophoresis of NotI digests of leptospiral DNA: a new rapid method of serovar identification. J Clin Microbiol. 1992;30:1696–702. doi: 10.1128/jcm.30.7.1696-1702.1992 1629323 PMC265366

[13] Majed Z, Bellenger E, Postic D, Pourcel C, Baranton G, Picardeau M. Identification of variable-number tandem-repeat loci in *Leptospira interrogans* sensu stricto. J Clin Microbiol. 2005;43:539–45. doi: 10.1128/JCM.43.2.539-545.2005 15695642 PMC548069

[14] Varni V, Ruybal P, Lauthier JJ, Tomasini N, Brihuega B, Koval A, et al. Reassessment of MLST schemes for *Leptospira* spp. typing worldwide. Infect Genet Evol. 2014;22:216–22. doi: 10.1016/j.meegid.2013.08.002 23932960

[15] Ahmed A, Thaipadungpanit J, Boonsilp S, Wuthiekanun V, Nalam K, Spratt BG, et al. Comparison of two multilocus sequence based genotyping schemes for *Leptospira* species. PLoS Negl Trop Dis 2011;5:e1374. doi: 10.1371/journal.pntd.0001374 22087342 PMC3210738

[16] Guglielmini J, Bourhy P, Schiettekatte O, Zinini F, Brisse S, Picardeau M. Genus-wide *Leptospira* core genome multilocus sequence typing for strain taxonomy and global surveillance. PLoS Negl Trop Dis. 2019;13:e0007374. doi: 10.1371/journal.pntd.0007374 31026256 PMC6513109

[17] Van CT, Thuy NT, San NH, Hien TT, Baranton G, Perolat P. Human leptospirosis in the Mekong delta, Viet Nam. Trans R Soc Trop Med Hyg. 1998;92:625–8. doi: 10.1016/s0035-9203(98)90787-8 10326104

[18] Rahman MHAA, Hairon SM, Hamat RA, Jamaluddin TZMT, Shafei MN, Idris N, et al. Seroprevalence and distribution of leptospirosis serovars among wet market workers in northeastern, Malaysia: a cross sectional study. BMC Infect Dis. 2018;18(569). doi: 10.1186/s12879-018-3470-5 30428852 PMC6236877

[19] Narkkul U, Thaipadungpanit J, Srisawat N, Rudge JW, Thongdee M, Pawarana R, et al. Human, animal, water source interactions and leptospirosis in Thailand. Sci Rep. 2021;11:3215. doi: 10.1038/s41598-021-82290-5 33547388 PMC7864926

[20] Garba B, Bahaman AR, Khairani-Bejo S, Zakaria Z, Mutalib AR. Retrospective Study of Leptospirosis in Malaysia. Ecohealth. 2017;14:389–98. doi: 10.1007/s10393-017-1234-0 28405850 PMC5486469

[21] Yanagihara Y, Villanueva SY, Yoshida S, Okamoto Y, Masuzawa T. Current status of leptospirosis in Japan and Philippines. Comp Immunol Microbiol Infect Dis 2007;30:399–413. doi: 10.1016/j.cimid.2007.05.003 17614131

[22] Victoriano AF, Smythe LD, Gloriani-Barzaga N, Cavinta LL, Kasai T, Limpakarnjanarat K, et al. Leptospirosis in the Asia Pacific region. BMC Infect Dis 2009;9:147. doi: 10.1186/1471-2334-9-147 19732423 PMC2749047

[23] Laras K, Cao BV, Bounlu K, Nguyen T, Olson JG, Thongchanh S, et al. The importance of leptospirosis in Southeast Asia. Am J Trop Med Hyg. 2002;67:278–86. doi: 10.4269/ajtmh.2002.67.278 12408667

[24] Kawaguchi L, Sengkeopraseuth B, Tsuyuoka R, Koizumi N, Akashi H, Vongphrachanh P, et al. Seroprevalence of leptospirosis and risk factor analysis in flood-prone rural areas in Lao PDR. Am J Trop Med Hyg. 2008;78:957–61. 18541776

[25] Mayxay M, Castonguay-Vanier J, Chansamouth V, Dubot-Pérès A, Paris DH, Phetsouvanh R, et al. Causes of non-malarial fever in Laos: a prospective study. Lancet Glob Health. 2013;1:e46–54. doi: 10.1016/S2214-109X(13)70008-1 24748368 PMC3986032

[26] Woods K, Nic-Fhogartaigh C, Arnold C, Boutthasavong L, Phuklia W, Lim C, et al. A comparison of two molecular methods for diagnosing leptospirosis from three different sample types in patients presenting with fever in Laos. Clin Microbiol Infect. 2017;S1198-743X:30579–7. doi: 10.1016/j.cmi.2017.10.017 29092789 PMC6125144

[27] Dittrich S, Rattanavong S, Lee SJ, Panyanivong P, Craig SB, Tulsiani SM, et al. Orientia, rickettsia, and *Leptospira* pathogens as causes of CNS infections in Laos: a prospective study. Lancet Glob Health. 2015;3:e104–12. doi: 10.1016/S2214-109X(14)70289-X 25617190 PMC4547322

[28] Syhavong B, Rasachack B, Smythe LD, Rolain JM, Roque-Afonso AM, Jenjaroen K, et al. The infective causes of hepatitis and jaundice amonst hospitalised patients in Vientiane, Laos. Transactions of the Royal Society of Tropical Medicine & Hygiene. 2010;104:475–83. 10.1016/j.trstmh.2010.03.002 PMC2896487 20378138

[29] Bounlu K, Insisiengmay S, Vanthanouvong K, Saykham, Widjaja S, Iinuma K, et al. Acute jaundice in Vientiane, Lao People’s Democratic Republic. Clin Infect Dis. 1998;27:717–21. doi: 10.1086/514948 9798023

[30] Boonsilp S, Thaipadungpanit J, Amornchai P, Wuthiekanun V, Bailey MS, Holden MTG, et al. A Single Multilocus Sequence Typing (MLST) Scheme for Seven Pathogenic *Leptospira* Species PLOS Negl Trop Dis. 2013;7:e1954. doi: 10.1371/journal.pntd.0001954 23359622 PMC3554523

[31] Phetsouvanh R, Phongmany S, Soukaloun D, Rasachak B, Soukhaseum V, Soukhaseum S, et al. Causes Of Community-Acquired Bacteremia And Patterns Of Antimicrobial Resistance In Vientiane, Laos. Am J Trop Med Hyg. 2006;75:978–85. 17124000 PMC2213713

[32] Johnson RC, Harris VG. Differentiation of pathogenic and saprophytic leptospires. J Bacteriol. 1967;94:27–31. doi: 10.1128/jb.94.1.27-31.1967 6027998 PMC251866

[33] Ellinghausen HC, McCullough WG. Nutrition of Leptospira pomona and growth of 13 other serotypes: fractionation of oleic albumin complex and a medium of bovine albumin and polysorbate 80. Am J Vet Res. 1965;26:45–51. 14266934

[34] Bourhy P, Collet L, Lernout T, Zinini F, Hartskeerl RA, van der Linden H, et al. Human *Leptospira* isolates circulating in Mayotte (Indian Ocean) have unique serological and molecular feature. J Clin Microbiol. 2012;50:307–11. doi: 10.1128/JCM.05931-11 22162544 PMC3264139

[35] Bourhy P, Herrmann-Storck C, Theodose R, Olive C, Nicolas M, Hochedez P, et al. Serovar diversity of pathogenic *Leptospira* circulating in the French West Indies. PLoS Negl Trop Dis. 2013;7:e2114. doi: 10.1371/journal.pntd.0002114 23516654 PMC3597474

[36] Wick RR, Judd LM, Gorrie CL, Holt KE. Unicycler: Resolving bacterial genome assemblies from short and long sequencing reads. PLoS Computational Biology. 2017;13:e1005595. doi: 10.1371/journal.pcbi.1005595 28594827 PMC5481147

[37] Wood DE, Lu J, Langmead B. Improved metagenomic analysis with Kraken 2. Genome Biol. 2019;20:257. doi: 10.1186/s13059-019-1891-0 31779668 PMC6883579

[38] Bankevich A, Nurk S, Antipov D, Gurevich AA, Dvorkin M, Kulikov AS, et al. SPAdes: A New Genome Assembly Algorithm and Its Applications to Single-Cell Sequencing. J Comp Biol. 2012;19:455–77. doi: 10.1089/cmb.2012.0021 22506599 PMC3342519

[39] Vaser R, Šikić M. Raven: a de novo genome assembler for long reads. Biorxiv. 2021. 10.1101/2020.08.07.242461

[40] Vaser R, Sović I, Nagarajan N, Šikić M. Fast and accurate de novo genome assembly from long uncorrected reads. Genome Res. 2017;27:737–46. doi: 10.1101/gr.214270.116 28100585 PMC5411768

[41] Walker BJ, Abeel T, Shea T, Priest M, Abouelliel A, Sakthikumar S, et al. Pilon: An Integrated Tool for Comprehensive Microbial Variant Detection and Genome Assembly Improvement. PLoS ONE. 2014;9:e112963. doi: 10.1371/journal.pone.0112963 25409509 PMC4237348

[42] Wang JR, Holt J, McMillan L, Jones CD. FMLRC: Hybrid long read error correction using an FM-index. BMC Bioinformatics. 2018;19:50. doi: 10.1186/s12859-018-2051-3 29426289 PMC5807796

[43] Vezzi F, Narzisi G, Mishra B. Reevaluating Assembly Evaluations with Feature Response Curves: GAGE and Assemblathons. PLoS ONE. 2012;7:e52210. doi: 10.1371/journal.pone.0052210 23284938 PMC3532452

[44] Li H, Handsaker B, Wysoker A, Fennell T, Ruan J, Homer N, et al. The Sequence Alignment/Map format and SAMtools. Bioinformatics. 2009;15:2078–9. doi: 10.1093/bioinformatics/btp352 19505943 PMC2723002

[45] Marçais G, Delcher AL, Phillippy AM, Coston R, Salzberg SL, Zimin A. MUMmer4: A fast and versatile genome alignment system. PloS Comput Biol. 2018;14:e1005944. doi: 10.1371/journal.pcbi.1005944 29373581 PMC5802927

[46] Hunt M, DeSilva N, Otto TD, Parkhill J, Keane JA, Harris SR. Circlator: automated circularization of genome assemblies using long sequencing reads. Genome Biol. 2015;16:294. doi: 10.1186/s13059-015-0849-0 26714481 PMC4699355

[47] Camacho C, Coulouris G, Avagyan V, Ma N, Papadopoulos J, Bealer K, et al. BLAST+: architecture and applications. BMC Bioinformatics 2009;10:421. doi: 10.1186/1471-2105-10-421 20003500 PMC2803857

[48] Grillová L, Oppelt J, Mikalová L, Nováková M, Giacani L, Niesnerová A, et al. Directly Sequenced Genomes of Contemporary Strains of Syphilis Reveal Recombination-Driven Diversity in Genes Encoding Predicted Surface-Exposed Antigens. Front Microbiol. 2019;10:1691. doi: 10.3389/fmicb.2019.01691 31417509 PMC6685089

[49] Contreras-Moreira B, Vinuesa P. GET_HOMOLOGUES, a versatile software package for scalable and robust microbial pangenome analysis. Appl Environ Microbiol. 2013;79:7696–701. doi: 10.1128/AEM.02411-13 24096415 PMC3837814

[50] Li Li L, Stoeckert CJJ, Roos DS. OrthoMCL: identification of ortholog groups for eukaryotic genomes. Genome Res. 2003;13:2178–89. doi: 10.1101/gr.1224503 12952885 PMC403725

[51] Huerta-Cepas J, Forslund K, Coelho LP, Szklarczyk D, Jensen LJ, vonMering C, et al. Fast Genome-Wide Functional Annotation through Orthology Assignment by eggNOG-Mapper. Mol Biol Evol. 2017;34:2115–22. doi: 10.1093/molbev/msx148 28460117 PMC5850834

[52] Stothard P, Wishart DS. Circular genome visualization and exploration using CGView. Bioinformatics 2005;21:537–9. doi: 10.1093/bioinformatics/bti054 15479716

[53] Tamura K, Stecher G, Peterson D, Filipski A, Kumar S. MEGA6: Molecular Evolutionary Genetics Analysis version 6.0. Mol Biol Evol 2013;30:2725–9. doi: 10.1093/molbev/mst197 24132122 PMC3840312

[54] MullerPlot: Generates Muller Plot from Population/Abundance/Frequency Dynamics Data. R package version 0.1.2 [Internet]. 2016.

[55] Merien F, Portnoi D, Bourhy P, Charavay F, Berlioz-Arthaud A, Baranton G. A rapid and quantitative method for the detection of *Leptospira* species in human leptospirosis. FEMS Microbiol Lett 2005;249: 139–147. doi: 10.1016/j.femsle.2005.06.011 16006065

[56] Stoddard RA, Gee JE, Wilkins PP, McCaustland K, Hoffmaster AR. Detection of pathogenic *Leptospira* spp. through TaqMan polymerase chain reaction targeting the LipL32 gene. DiagnMicrobiol Infect Dis 2009;64:247–55. doi: 10.1016/j.diagmicrobio.2009.03.014 19395218

[57] Ahmed N, Devi SM, Valverde Mde L, Vijayachari P, Machang’u RS, Ellis WA, et al. Multilocus sequence typing method for identification and genotypic classification of pathogenic *Leptospira* species. Ann Clin Microbiol Antimicrob 2006;5:28. doi: 10.1186/1476-0711-5-28 17121682 PMC1664579

[58] Ahmed A, Engelberts MF, Boer KR, Ahmed N, Hartskeerl RA. Development and validation of a real-time PCR for detection of pathogenic *Leptospira* species in clinical materials. PLoS One. 2009;4:e7093. doi: 10.1371/journal.pone.0007093 19763264 PMC2740861

[59] Thaipadungpanit J, Wuthiekanun V, Chierakul W, Smythe LD, Petkanchanapong W, Limpaiboon R, et al. A Dominant Clone of *Leptospira interrogans* Associated with an Outbreak of Human Leptospirosis in Thailand. PLoS Negl Trop Dis. 2007;1(1):e56. doi: 10.1371/journal.pntd.0000056 . 17989782 PMC2041815

[60] Murray GL. The molecular basis of leptospiral pathogenesis. Curr Top Microbiol Immunol. 2015;387:139–85. doi: 10.1007/978-3-662-45059-8_7 25388135

[61] Qin QL, Xie BB, Zhang XY, Chen XL, Zhou BC, Zhou J, et al. A proposed genus boundary for the prokaryotes based on genomic insights. J Bacteriol. 2014;196:2210–5. doi: 10.1128/JB.01688-14 24706738 PMC4054180

[62] Smith S, Kennedy BJ, Dermedgoglou A, Poulgrain SS, Paavola MP, Minto TL, et al. A simple score to predict severe leptospirosis. PLoS Negl Trop Dis. 2019;13:e0007205. doi: 10.1371/journal.pntd.0007205 30759081 PMC6391019

[63] Herrmann-Storck C, Saint-Louis M, Foucand T, Lamaury I, Deloumeaux J, Baranton G, et al. Severe leptospirosis in hospitalized patients, Guadeloupe. Emerg Infect Dis. 2010;16(2):331–4. Epub 2010/02/02. doi: 10.3201/eid1602.090139 . 20113574 PMC2957992

[64] Hinjoy S, Kongyu S, Doung-Ngern P, Doungchawee G, Colombe SD, Tsukayama R, et al. Environmental and Behavioral Risk Factors for Severe Leptospirosis in Thailand. Trop Med Infect Dis. 2019;4:79. doi: 10.3390/tropicalmed4020079 31100812 PMC6631942

[65] Daher E, Zanetta DM, Cavalcante MB, Abdulkader RC. Risk factors for death and changing patterns in leptospirosis acute renal failure. Am J Trop Med Hyg. 1999;61(4):630–4. Epub 1999/11/05. doi: 10.4269/ajtmh.1999.61.630 . 10548299

[66] Stoddard RA, Bui D, Haberling DL, Wuthiekanun V, Thaipadungpanit J, Hoffmaster AR. Viability of *Leptospira* isolates from a human outbreak in Thailand in various water types, pH, and temperature conditions. Am J Trop Med Hyg 2014;9:1020–2. 10.4269/ajtmh.13-0748 PMC4228868 25200260

[67] Cosson JF M. P, Mielcarek M, Tatard C, Chaval Y, Suputtamongkol Y, et al. Epidemiology of *Leptospira* transmitted by rodents in southeast Asia. PLoS Negl Trop Dis. 2014;8:e2902. doi: 10.1371/journal.pntd.0002902 24901706 PMC4046967

[68] Brockhurst MA, Harrison E, Hall JPJ, Richards T, McNally A, MacLean C. The ecology and evolution of Pangenomes. Curr Biol. 2019;29:R1094–R103. doi: 10.1016/j.cub.2019.08.012 31639358

[69] Picardeau M. Virulence of the zoonotic agent of leptospirosis: still terra incognita? Nat Rev Microbiol 2017;15:297–307. doi: 10.1038/nrmicro.2017.5 28260786

[70] Grillová L, Angermeier H, Levy M, Giard M, Lastère S, Picardeau M. Circulating genotypes of *Leptospira* in French Polynesia: An 9-year molecular epidemiology surveillance follow-up study. PLoS Negl Trop Dis. 2020;14:e0008662. doi: 10.1371/journal.pntd.0008662 32986693 PMC7544043

[71] Chakraborty A, Miyahara S, Villanueva SY, Saito M, Gloriani NG, Yoshida S. A novel combination of selective agents for isolation of *Leptospira* species. Microbiol Immunol. 2011;55:494–501. doi: 10.1111/j.1348-0421.2011.00347.x 21545510

[72] Hornsby RL, Alt DP, Nally JE. Isolation and propagation of leptospires at 37°C directly from the mammalian host. Sci Rep. 2020;10:9620. doi: 10.1038/s41598-020-66526-4 32541841 PMC7296004

