# Supplementary material for: Genetic diversity of Leptospira isolates in Lao PDR and genome analysis of an outbreak strain
Source: PLoS Negl Trop Dis. 2021 Dec 28;15(12):e0010076. doi: 10.1371/journal.pntd.0010076 (PMC8746763; doi:10.1371/journal.pntd.0010076)
Supplement: S7 Table — (DOCX) [file pntd.0010076.s007.docx]

**S7 Table : The most frequently used diagnostic and/or typing PCR assays**

| Locus | Primer | Sequence (5'-3') | Size of the PCR product | Reference |
| --- | --- | --- | --- | --- |
| *lfb*1 | LFB1-F | CATTCATGTTTCGAATCATTTCAAA |  | [1] |
|  | LFB1-R | GGCCCAAGTTCCTTCTAAAAG | 335 bp |  |
| *lipL*32 | LipL32-45F | AAGCATTACCGCTTGTGGTG |  |  |
|  | LipL32-286R | GAACTCCCATTTCAGCGATT | 242 bp | [2] |
| *sec*Y | SecYIVF | GCGATTCAGTTTAATCCTGC |  |  |
|  | SecYIVR | GAGTTAGAGCTCAAATCTAAG | 203 bp | [3] |
|  | secY-F | ATGCCGATCATTTTTGCTTC |  |  |
|  | secY-R | CCGTCCCTTAATTTTAGACTTCTTC | 549 bp | [4] |

**Reference**

1. Merien F, Portnoi D, Bourhy P, Charavay F, Berlioz-Arthaud A, Baranton G. A rapid and quantitative method for the detection of Leptospira species in human leptospirosis. FEMS Microbiol Lett 2005;249: 139-147.

2. Stoddard RA, Gee JE, Wilkins PP, McCaustland K, Hoffmaster AR. Detection of pathogenic Leptospira spp. through TaqMan polymerase chain reaction targeting the LipL32 gene. DiagnMicrobiol Infect Dis 2009;64:247-55.

3. Ahmed A, Engelberts MF, Boer KR, Ahmed N, Hartskeerl RA. Development and validation of a real-time PCR for detection of pathogenic leptospira species in clinical materials. PLoS One. 2009;4:e7093.

4. Ahmed N, Devi SM, Valverde Mde L, Vijayachari P, Machang'u RS, Ellis WA, et al. Multilocus sequence typing method for identification and genotypic classification of pathogenic Leptospira species. Ann Clin Microbiol Antimicrob 2006;5:28.
