# Supplementary material for: Genetic diversity of Leptospira isolates in Lao PDR and genome analysis of an outbreak strain
Source: PLoS Negl Trop Dis. 2021 Dec 28;15(12):e0010076. doi: 10.1371/journal.pntd.0010076 (PMC8746763; doi:10.1371/journal.pntd.0010076)
Supplement: S1 Fig — Gene frequency plot showing the frequency of genes within the Leptospira strains. Analyses done with GET_HOMOLOGUES showing the U-shaped distribution of pan-genome from the three groups of strains: L. interrogans CG272 strains (CG272), L. interrogans non-CG272 strains (Others) and all the Leptospira strains (complete). (DOCX) [file pntd.0010076.s008.docx]

**S1 Fig. Pan-genome distribution in four categories (cloud, shell, soft core and core) for *Leptospira* strains from Laos.**

Gene frequency plot showing the frequency of genes within the *Leptospira* strains. Analyses done with GET_HOMOLOGUES showing the U-shaped distribution of pan-genome from the three groups of strains : *L. interrogans* CG272 strains (CG272), *L. interrogans* non-CG272 strains (Others) and all the *Leptospira* strains (complete).
