# Supplementary material for: Genetic diversity of Leptospira isolates in Lao PDR and genome analysis of an outbreak strain
Source: PLoS Negl Trop Dis. 2021 Dec 28;15(12):e0010076. doi: 10.1371/journal.pntd.0010076 (PMC8746763; doi:10.1371/journal.pntd.0010076)
Supplement: S2 Fig — Minimum spanning tree was created using GrapeTree for visualization of core genomic relationships [1]. Every tree node represents a core genome of a single sample, the cgMLST clonal groups are indicated by the numbers inside the tree nodes and the geographic origin is determined by colors. Strains are listed in S6 Table. The base layer of the map is from outline-world-map.com. (DOCX) [file pntd.0010076.s009.docx]

**S2 Fig.** Core genes of all available L. interrogans strains isolated from human patients in Southeast Asia (n=81) during 1905 – 2017. Minimum spanning tree was created using GrapeTree for visualization of core genomic relationships [1]. Every tree node represents a core genome of a single sample, the cgMLST clonal groups are indicated by the numbers inside the tree nodes and the geographic origin is determined by colors. Strains are listed in S6 Table. The base layer of the map is from outline-world-map.com.

Reference

1. Zhou Z, Alikhan NF, Sergeant MJ, Luhmann N, Vaz C, Francisco AP, et al. GrapeTree: visualization of core genomic relationships among 100,000 bacterial pathogens. Genome Res. 2018;(28):1395-404.
